# Health Benefits and Side Effects of Short-Chain Fatty Acids

**DOI:** 10.3390/foods11182863

**Published:** 2022-09-15

**Authors:** Ruo-Gu Xiong, Dan-Dan Zhou, Si-Xia Wu, Si-Yu Huang, Adila Saimaiti, Zhi-Jun Yang, Ao Shang, Cai-Ning Zhao, Ren-You Gan, Hua-Bin Li

**Affiliations:** 1 Guangdong Provincial Key Laboratory of Food, Nutrition and Health, Department of Nutrition, School of Public Health, Sun Yat-Sen University, Guangzhou 510080, China; 2 School of Chinese Medicine, Li Ka Shing Faculty of Medicine, The University of Hong Kong, Hong Kong 999077, China; 3 Department of Clinical Oncology, Li Ka Shing Faculty of Medicine, The University of Hong Kong, Hong Kong 999077, China; 4 Research Center for Plants and Human Health, Institute of Urban Agriculture, Chinese Academy of Agricultural Sciences, National Agricultural Science & Technology Center, Chengdu 610213, China

**Keywords:** SCFAs, inflammation, cardiovascular disease, obesity, diabetes mellitus, cancer

## Abstract

The gut microbiota and their metabolites could play an important role in health and diseases of human beings. Short-chain fatty acids (SCFAs) are mainly produced by gut microbiome fermentation of dietary fiber and could also be produced by bacteria of the skin and vagina. Acetate, propionate, and butyrate are three major SCFAs, and their bioactivities have been widely studied. The SCFAs have many health benefits, such as anti-inflammatory, immunoregulatory, anti-obesity, anti-diabetes, anticancer, cardiovascular protective, hepatoprotective, and neuroprotective activities. This paper summarizes health benefits and side effects of SCFAs with a special attention paid to the mechanisms of action. This paper provides better support for people eating dietary fiber as well as ways for dietary fiber to be developed into functional food to prevent diseases.

## 1. Introduction

**Figure 1:**
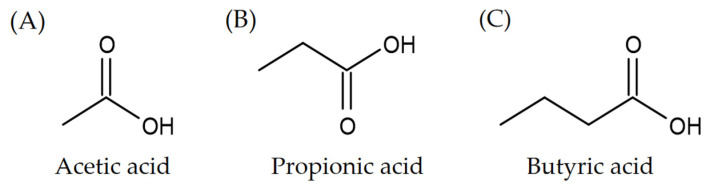
(**A**–**C**) The chemical structures of three major SCFAs.

Short-chain fatty acids (SCFAs) are mainly metabolites of dietary fiber and protein in gut [1,2], and they could also be produced by bacteria of the skin and vagina [3,4]. Dietary fibers are main sources of SCFAs, and they can be classified as soluble (such as pectins and inulin) and insoluble dietary fibers (such as various forms of resistant starches) [5]. The SCFAs have less than six carbon atom numbers and mainly include acetate, propionate, butyrate, pentanoate, malonate, and so on [6]. Among them, acetate, propionate, and butyrate are three major SCFAs, which account for 90% of SCFAs produced by gut microbiota [6]. The chemical structures of three SCFAs are shown in Figure 1. The SCFAs are produced primarily in the cecum and proximal colon, and their concentrations decline from proximal to the distal colon as the substrates used for fermentation are exhausted gradually [7]. Several factors affected the production of SCFAs, such as substrate source, composition of gut microbes, colonic pH value, gut transit time, and site of substrate fermentation [8,9]. For example, a study showed that resistant starch 5 (amylose–lipid complexes) produced more butyric acid than resistant starch 2 (nongelatinized native starch granule) and resistant starch 3 (retrograded starch) [10]. Another study indicated that low colonic pH value promoted butyrate production and increased populations of butyrate-producing bacteria [11]. In addition, host physiology, such as intestinal environment, microbe–host interaction, and even social stress, could affect SCFA production [12]. For instance, a study found that stress exposure reduced the levels of colonic SCFAs in mice through modulating the gut microbiota, such as decreasing the SCFAs producing genera *Anaerostipes*, *Butyricicoccus*, *Coprococcus,* and *Parabacteroides*, as well as increasing the abundance of *Odoribacter* [13]. Furthermore, more than 90% of SCFAs are absorbed from the intestinal cavity and metabolized by colonocytes or liver [8]. Butyrate is an important energy source for colonocytes, and therefore only a small amount of butyrate reaches the hepatic system, while other absorbed SCFAs that have not been metabolized by colonocytes, particularly acetate and propionate, could reach the liver via the portal vein [7,14]. The liver is a major site for the metabolism of SCFAs in humans, since approximately 40% of acetate and 80% of propionate in portal vein are taken up and metabolize by the liver [15]. Moreover, a small portion of SCFAs in the rectum could bypass the liver and pass directly into the systemic circulation via the internal iliac vein [7,8]. 

In recent years, many studies have proven that intestinal microbiota and their metabolites play a vital role in human health [16,17]. The gut microbiota-derived SCFAs have shown a variety of biological effects on the host, such as anti-inflammatory, immunoregulatory, anti-obesity, anti-diabetes, anticancer, cardiovascular protective, hepatoprotective, and neuroprotective effects [6,18,19]. The role of SCFAs in human health and diseases has become a research hotspot. This narrative review collects the literature from the Web of Science Core Collection and PubMed databases in the past five years based on keywords in the title and abstract, including short-chain fatty acids, SCFAs, acetic acid, acetate, propionic acid, propionate, butyric acid, butyrate, isobutyric acid, isobutyrate, valeric acid, valerate, hexanoic acid, and hexanoate, and summarizes the bioactivities of SCFAs with special attention paid to their mechanisms of action.

## 2. The Bioactivities of SCFAs

**Figure 2:**
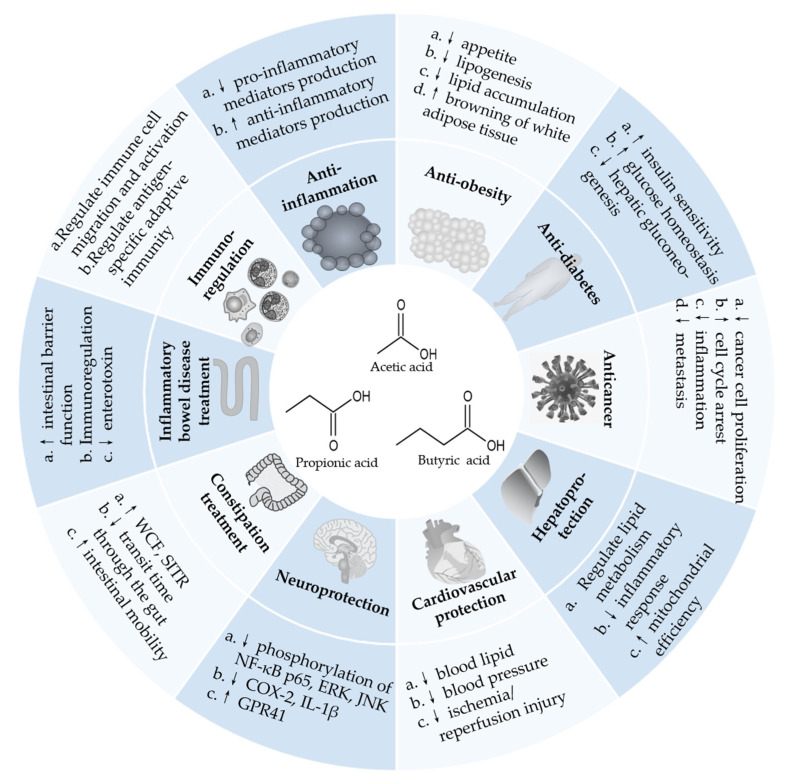
Health benefits and the related mechanisms of SCFAs. ↓ represents decrease, and ↑ represents increase. Abbreviation: COX-2, cyclooxygenase-2; GPR41, G-protein-coupled receptor 41; IL, interleukin; NF-κB, nuclear factor-kappa B; SITR, small intestinal transit rate; WCF, water content of feces.

The health benefits of SCFAs have been widely studied, and the mechanisms of action have been explored (Table 1, Table 2 and Table 3 and Figure 2), which are summarized and discussed in detail below. In each section, the results of epidemiological studies are first described, then the results of preclinical studies are discussed, and the results of clinical studies (if any) are finally elaborated. 

### 2.1. Anti-Inflammatory Activity

Inflammation is related to the occurrence and development of many diseases. In recent years, many studies demonstrated that SCFAs could reduce the production of inflammatory factors through several signaling pathways. The SCFAs attenuated the inflammatory response by decreasing the production of pro-inflammatory mediators and enhancing the production of anti-inflammatory mediators. A study indicated that propionate and butyrate alleviated the inflammation in cells by inhibiting the expressions of interleukin (IL)-6, reactive oxygen species (ROS), as well as enhancing the expressions of IL-10 [20]. Besides, it was reported that butyrate attenuated the inflammation induced by lipopolysaccharide (LPS) via up-regulating IL-10 in septic shock [21]. Additionally, the evidence showed that acetate effectively resolved neutrophilic inflammation via inducing caspase-dependent apoptosis of neutrophils, decreasing the activity of nuclear factor-kappa B (NF-κB) and enhancing the production of anti-inflammatory mediators, such as IL-10, transforming growth factor-β (TGF-β), and annexin A1 [22]. In the LPS-treated neonatal mice model, pulmonary inflammation and oxidative stress were reduced by sodium propionate; in the LPS-treated human pulmonary microvascular endothelial cells (HPMECs) model, sodium propionate not only accelerated Nrf2 nuclear translocation, protected cells, and promoted angiogenesis, but also reduced inflammatory response via the NF-κB pathway [23]. Moreover, one in vivo study showed that propionate interfered with the production and migration of inflammatory mediators [24]. Furthermore, some SCFAs had complex bidirectional regulatory properties. It was found that the level of acetate negatively correlated with the pro-inflammatory biomarker interferon-γ (IFN-γ), while the levels of butyrate and valerate positively correlated with IFN-γ and tumor necrosis factor-α (TNF-α) [25]. 

In summary, SCFAs exhibit good anti-inflammatory activity, and the main mechanisms of action include inhibiting the production of pro-inflammatory mediators, such as IL-6 and TNF-α, as well as enhancing the production of anti-inflammatory mediators, such as IL-10, TGF-β, and annexin A1. In the future, more in vivo studies are needed to prove the bidirectional regulation of SCFAs on inflammatory factors and discover its mechanisms.

### 2.2. Immunoregulatory Activity

More and more studies have shown that gut microbiota play a vital role in the host’s immune system, and the effects are mainly carried out by producing metabolites, such as SCFAs [26]. The SCFAs could regulate the function of innate immune cells to participate in the immune system, such as dendritic cells. In an ovalbumin-induced allergic mice model, dietary supplement with SCFAs could prevent the exacerbation of lung inflammation induced by vancomycin, via attenuating dendritic cells migration and activation [27]. The SCFAs could also regulate the differentiation and function of T and B cells, and then mediate antigen-specific adaptive immunity. For example, SCFAs induced the production of IL-22 by CD4^+^ T cells through binding G-protein receptor 41 (GPR41) and inhibiting histone deacetylase (HDAC) [28]. Besides, butyrate promoted the production of IL-22 via increasing hypoxia-inducible factor (HIF) 1α binding to the *Il22* promoter through histone modification [28]. For B cells, SCFAs promoted B cell differentiation by increasing acetyl-coenzyme A (acetyl-CoA), oxidative phosphorylation, glycolysis, and fatty acid synthesis [26]. Moreover, as efficient HDAC inhibitors, SCFAs could stimulate B cell differentiation via boosting the expression of B cell differentiation-related genes, such as *Xbp-1*, *Aicda,* and *Prdm1* [26]. The SCFAs could also decrease circulating immunoglobulin (Ig) E level [27]. Furthermore, SCFAs could promote murine and human B10 cell generation via inhibiting HDAC [29].

In short, SCFAs participate in the function of immune system via reducing the migration and activation of dendritic cells to relieve allergy, as well as promoting T and B cells’ differentiation to regulate antigen-specific adaptive immunity. The immunoregulatory action of SCFAs are mainly achieved by directly binding SCFA-specific G-protein-coupled receptor (such as GPR41) on the cell surface and entering cells to regulate cell metabolism and inhibit HDAC.

### 2.3. Anti-Obesity Activity

Obesity is a metabolic disorder and mainly caused by an imbalance between energy intake and expenditure [19,30]. Recently, many studies indicated that SCFAs played a vital role in the management of obesity. For example, one epidemiological study suggested that human milk SCFAs exerted a protective effect against excess weight gain in infants [31].

SCFAs play an important part in obesity and energy metabolism by regulating the appetite. For example, a study showed that the mechanism of SCFAs suppressing food intake was related to vagal afferent, and the efficacy was butyrate > propionate > acetate. Moreover, butyrate exerted an anorexigenic effect through activating vagal afferent neurons and their projection sites, such as nucleus tractus solitaries (NTS) neurons, and directly increasing Ca^2+^ concentration in nodose ganglion neurons (NGNs) [32]. Besides, serum glucagon-like peptide 1 (GLP-1), peptide YY (PYY), and leptin participated in the short-term signal of satiety transferring to the appetite center of the brain. A 28-day experiment of pigs showed that acetate and propionate increased the concentrations of serum GLP-1, PYY, and leptin, and then reduced the appetite [33]. In addition, SCFAs reduced appetite and fat accumulation via modulating relevant genes and hormones, such as mitochondrial transcription factor A, tumor necrosis factor receptor superfamily member 9, cytochrome-C oxidase IV, and free fatty acid receptor 2 [34]. 

The SCFAs could also exert an anti-obesity effect via other different pathways. It was reported that SCFAs could restrain lipogenesis [35]. The SCFAs mediated the suppression of de novo lipogenesis in male rats by suppressing hepatic acetyl-CoA carboxylase-1(*Acc1*) expression [35]. Furthermore, propionate repressed the triglyceride (TG) accumulation via modulating the expression levels of PPARα-responsive genes, such as carnitine palmitoyl transferase II (*CPTII*) and trifunctional protein alpha (*TFPα*) [36]. Another study suggested that SCFAs could protect against high-fat diet-induced obesity and suppress lipid synthesis [37]. Besides, propionate reduced obesity-associated metabolic disturbances via decreasing the hepatic TG and increasing odd-chain fatty acids (OCFAs) formation [38]. Additionally, acetate could decrease lipid accumulation, inhibit white adipose tissue lipolysis and induce browning of white adipose tissue, which could reduce the body adiposity by increasing thermogenesis [39]. Furthermore, a randomized, placebo-controlled, single-blind crossover study showed that inulin-propionate ester (10 g/day) significantly decreased the striatal blood oxygen level-dependent signal, high energy food picture appeal, and energy intake at an ad libitum meal [40].

**Figure 3:**
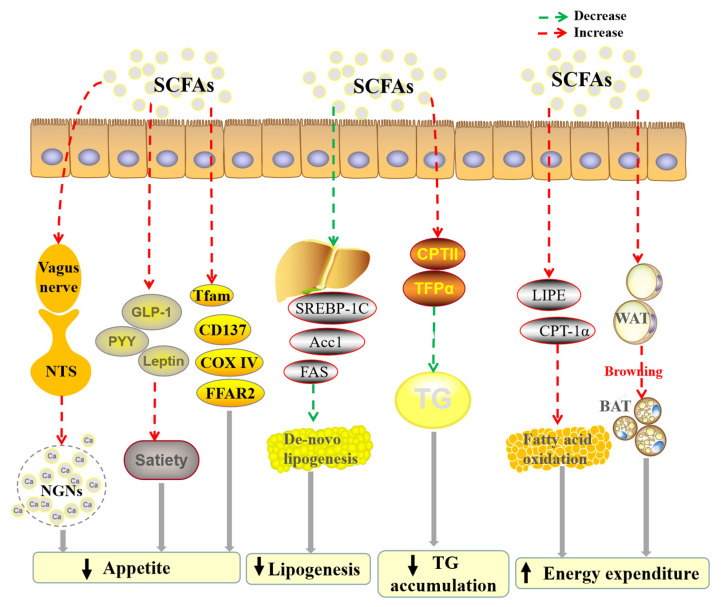
The main effects and mechanisms of SCFAs on obesity. ↓ represents decrease, and ↑ represents increase. Abbreviation: Acc1, acetyl-CoA carboxylase-1; BAT, brown adipose tissue; CD137, tumor necrosis factor receptor superfamily member 9; COX IV, cytochrome-C oxidase IV; CPT, carnitine palmitoyl transferase; FAS, fatty acid synthase; FFAR2, free fatty acid receptor 2; GLP-1, glucagon-like peptide 1; LIPE, lipase hormone sensitive; NTS, nucleus tractus solitaries; PYY, peptide YY; SCFA, short-chain fatty acid; SREBP-1C, sterol-regulatory element-binding protein 1C; Tfam, mitochondrial transcription factor A; TFPα, trifunctional protein alpha; WAT, white adipose tissue.

In other words, SCFAs have an obvious anti-obesity effect, and the mechanisms involve suppressing appetite, restraining the lipogenesis, and inducing browning of white adipose tissue (Figure 3). Furthermore, the effect of SCFAs on lipid accumulation needs to be confirmed by more studies.

### 2.4. Cardio-Protective Activity

Cardiovascular disease (CVD) is a chronic non-communicable disease with high morbidity and mortality on a global scale [41]. SCFAs have good protective effects on cardiovascular system [42], and the related mechanisms of action are discussed below. 

SCFAs could protect cardiovascular system by decreasing atherosclerosis. For example, a study showed that butyrate inhibited the progression of diet-induced atherosclerosis by decreasing intestinal cholesterol absorption via regulating related transporters expression, such as Niemann-Pick C1-like 1 (Npc1l1, a major intestinal cholesterol transporter) and ATP-binding cassette (ABC) transporters G5 and G8 [43]. Moreover, propionate reduced intestinal cholesterol absorption and aortic atherosclerotic lesion area via increasing levels of regulatory T (Treg) cell and IL-10 and reduced the expression of Npc1l1 [44]. Moreover, the elevation of plasma total cholesterol (TC) is an important risk factor for atherosclerosis. The SCFAs significantly reduced plasma TC via enhancing fecal excretion of bile acids and up-regulating the gene expressions of sterol-regulatory element-binding protein 2 (SREBP2), low-density lipoprotein (LDL) receptor, and cholesterol 7 alpha-hydroxylase (CYP7A1) in the liver [45]. Furthermore, a randomized, double-blind clinical trial suggested that propionate intake (500 mg, twice daily) significantly reduced levels of LDL and non-high-density lipoprotein cholesterol, which were effective targets for atherosclerotic CVD prevention [44].

SCFAs could exert a cardiovascular protective effect by inhibiting hypertension. For instance, one study suggested that acetate, butyrate, and propionate could reduce blood pressure, and acetate showed the most powerful antihypertensive effect among these three SCFAs [46]. Moreover, acetate kept the balance of vasoconstriction and vasodilation shifts through up-regulation of SCFAs receptors, Olfr78, GPR41, and GPR43, in which way to prevent high-fructose diet-induced hypertension [47]. Besides, an in vivo study showed that propionate alleviated cardiac hypertrophy, fibrosis, vascular dysfunction, and hypertension in both wild-type NMRI and ApoE^−/−^ mice models, and the mechanisms mainly depended on Treg cells [48].

The evidence indicated that SCFAs had protective effects against ischemia/reperfusion-related injuries (IRI). Butyrate significantly improved myocardial IRI via the gut–brain neural circuit and might be mediated by the paraventricular nucleus (PVN)-superior cervical ganglion (SCG) sympathetic pathway [49]. Besides, acetate, butyrate, and propionate repaired the myocardial infarction (MI) impairment by reducing the infiltration of CX3CR1+ monocytes to the peri-infarct zone after MI and played an important part in maintaining host immune composition [50].

In summary, SCFAs could be a potential therapeutic strategy to prevent and manage CVD through reducing blood lipid and blood pressure, alleviating IRI, and repairing MI injury.

### 2.5. Hepatoprotective Activity

Liver diseases are generally divided into non-viral liver diseases and viral liver diseases [51]. Recent studies have found that gut microbiota and its metabolites, such as SCFAs, could prevent and manage several liver diseases, particularly non-viral liver diseases [52,53]. An epidemiology study found that fecal SCFAs were negatively correlated with cirrhosis disease severity [54].

Non-viral liver diseases mainly involve alcoholic liver disease (ALD), nonalcoholic fatty liver disease (NAFLD), and drug- or pollutant-induced liver injury. ALD could be caused by a long-term heavy alcohol intake, which involved alcoholic hepatitis, fibrosis, and cirrhosis [55]. A study found that propionate alleviated the ethanol-induced hepatic steatosis and enhanced hepatic function through maintaining the intestinal epithelial barrier function and inhibiting hepatic toll-like receptor 4 (TLR4)-NF-κB pathway [56]. The occurrence of NAFLD was closely related to intestinal flora disturbance, and gut microbiota-derived SCFAs could be a valuable strategy for preventing and treating NAFLD [57]. The SCFAs could regulate mucus secretion, microbial homeostasis, and intestinal epithelial tight junction to reduce the spread of intestinal endotoxin to the liver, thereby reducing the oxidative pressure and the level of inflammation in the liver and then delaying the development of NAFLD [58]. In addition, SCFAs changed the intestinal micro-ecology to protect the gut barrier, which could slow down the development of NAFLD-related diseases [58]. Another study suggested that SCFAs could inhibit hepatic steatosis by activating AMPK and PPAR signaling pathways and down-regulating the expression of genes related to lipid synthesis, such as sterol-regulatory element-binding protein 1 (*SREBP-1*), *FAS*, stearoyl-CoA desaturase 1 (*SCD1*), *ACC1*, and liver X receptor (*LXR*) [59]. Furthermore, pectin, Jaboticaba berry peel, and fu instant tea could alleviate fatty liver disease by regulating intestinal SCFAs [60,61,62]. As for drug- or pollutant-induced liver injury, SCFAs also exerted hepatoprotective effects. Cytochrome p450 (CYP) maturation in the liver is important for metabolic activity and xenobiotic detoxification. An in vitro study showed that the mixture of acetate, propionate, and butyrate increased the expression of CYP3A4 and ALB in human-induced pluripotent stem cell-derived liver organoids, which improved the hepatic maturation and enhanced metabolic activity and xenobiotic detoxification [63]. Furthermore, it was reported that acetate reduced serum levels of aspartate aminotransferase and alkaline phosphatase, which indicated that it improved hepatic function. Meanwhile, acetate increased mitochondrial efficiency and adenosine triphosphate production [39]. 

In brief, SCFAs play a positive role in non-viral liver diseases. The mechanisms of action involved maintaining the intestinal epithelial barrier, regulating the lipid metabolism and inflammatory response in liver, increasing mitochondrial efficiency, and promoting CYP maturation. 

### 2.6. Anti-Diabetic Activity

Diabetes mellitus is characterized by hyperglycemia caused by decreasing insulin secretion or insulin resistance, and 592 million people will have diabetes mellitus by the year 2035 worldwide according to the International Diabetes Mellitus Federation prediction [64,65]. The effects of SCFAs on diabetes mellitus have been widely studied. A microbiome-wide association study on large population cohorts showed that butyrate and acetate had a causal relationship with type 2 diabetes using bidirectional Mendelian randomization analyses [66]. 

Propionate attenuated high-fat diet-induced insulin resistance and improved insulin sensitivity, and the mechanism of action might relate to stimulating OCFA production [38]. Besides, acetate and propionate improved insulin sensitivity and glucose tolerance [67]. In addition, the combination of acetate and propionate effectively improved insulin sensitivity in high-fat diet-fed mice via reducing inflammation through decrease of T helper 1 (Th1) and Th2 cells and increase of Treg cells in the spleen and mesenteric lymph node [67]. Moreover, butyrate could promote the growth of intestinal epithelial cells, strengthen the intestinal tight connection, and regulate the activities of gut microbiota and immune cells, in which way to maintain the gut barrier and ultimately prevent and treat type 1 diabetes mellitus [58]. Several studies also showed that oral and dietary supplementation of butyrate, as well as human acetate colonic infusions and vinegar administrations, could prevent high-fat diet-induced insulin resistance and improve glucose homeostasis [68,69]. Additionally, propionate could activate AMP-activated protein kinase (AMPK) by binding GPR43 and increasing intracellular Ca^2+^. Besides, propionate suppressed hepatic gluconeogenesis via down-regulating gluconeogenic enzymes, such as glucose-6-phosphatase (G6Pase) and phosphoenolpyruvate carboxykinase (PEPCK), through activation of AMPK [70]. Furthermore, acylated starch had a greater effect on the improvement of type 2 diabetes indexes compared to native resistant starch, such as fasting blood glucose, serum insulin level, and amino acid metabolism [71]. In addition, a randomized clinical trial based on 29 overweight/obese individuals suggested that after the Mediterranean diet intervention for 8 weeks, postprandial plasma butyric acid incremental area under the curve (IAUC) was significantly increased, which was negatively correlated with plasma insulin IAUC and oral glucose insulin sensitivity [72]. 

Overall, SCFAs could prevent and manage diabetes mellitus via increasing insulin sensitivity, improving glucose homeostasis, and suppressing hepatic gluconeogenesis. Furthermore, more high-quality clinical studies are also needed to determine the effect of SCFAs on glycemic control and diabetic mellitus.

### 2.7. Effects on Inflammatory Bowel Diseases

**Figure 4:**
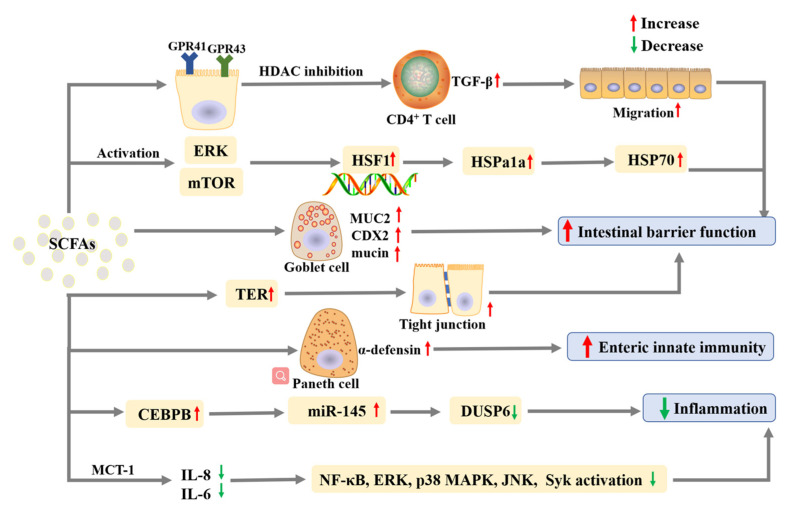
The main effects and mechanisms of SCFAs on inflammatory bowel diseases. Abbreviation: CEBPB, CCAAT enhancer-binding protein beta; DUSP6, dual-specificity phosphatase 6; ERK, extracellular signal-regulated kinase; GRP, G-protein receptor; HDAC, histone deacetylase; HSF, heat shock factor; HSP, heat shock proteins; IL, interleukin; JNK, c-Jun N-terminal kinase; MAPK, p38 mitogen-activated protein kinase; MCT-1, monocarboxylate transporter 1; miR-145, microRNA-145; mTOR, mechanistic target of rapamycin; NF-κB, nuclear factor-kappa B; SCFAs, short-chain fatty acid; Syk, spleen tyrosine kinase; TGF-β, transforming growth factor-β.

The inflammatory bowel diseases (IBDs) are complex immune-mediated diseases characterized by chronic inflammation in the gastrointestinal tract, and caused by the interaction among genetic, immunologic, microbial, and environmental factors [13]. SCFAs are believed to play a beneficial role in human gut health and prevent IBDs via different pathways [73], and the mechanisms of action are shown in Figure 4. The gut epithelium consists of a layer of constantly renewing epithelial cells and becomes the first line of defense against enteric infection [74]. A study showed that propionate promoted intestinal epithelial cell migration and increased cell speed and persistence in a HDAC inhibition, GPR43, and the signal transducer and activator of transcription 3 (STAT3) in a dependent manner [74]. Another study showed that butyrate promoted intestinal integrity and homeostasis via affecting metabolism, intestinal barrier function, and transporter expression [75]. Heat shock proteins (HSP) play a crucial role in maintaining and protecting the integrity of intestinal epithelial cells. Propionate contributed to intestinal homeostasis via increasing the level of *Hspa1a* (a major transcript of HSP70), up-regulating HSP70, and phosphorylating heat shock factor 1 [76]. Moreover, the transcriptional activation of HSP70 was proven to be related to the activation of mitogen-activated protein kinase (MEK)/ extracellular signal-regulated kinase (ERK) and mTOR pathways, as well as the inhibition of HDAC [76]. Besides, acetate increased the expression of MUC2 and CDX2, as well as the production of mucin proteins, in mucus-secreting colon epithelial cells (HT29-MTX), which indicated that it could improve the intestinal epithelium protective function [77]. SCFAs in an optimal dose could modulate the structure of gut microbiota, regulate the activities of immune cells and intestinal epithelial cells via regulating the gene expression of intestinal cells in an HDAC-dependent way, and subsequently improve the gut barrier function [58]. It was reported that acetate, propionate, and butyrate not only increased transepithelial electrical resistance (TER) and improved the formation of tight junction, but also inhibited the activation of NLRP3 inflammasome and autophagy induced by LPS to protect intestinal barrier function [78]. The SCFAs contributed to intestinal homeostasis via regulating the immune system and reducing inflammation. For example, butyric acid elicited α-defensin secretion by Paneth cells and improved enteric innate immunity through potent microbicidal activities, which contributed to intestinal homeostasis [79]. Besides, propionate repressed IL-17- and IL-22-producing γδ T cells to relieve intestinal inflammation [80]. Moreover, SCFAs transported by monocarboxylate transporter (MCT)-1 could suppress inflammatory responses in Caco-2 cells induced by TNF-α via decreasing IL-8 and IL-6 expression levels and inhibiting the activation of NF-κB, ERK, p38 mitogen-activated protein kinase (MAPK), c-Jun N-terminal kinase (JNK), and spleen tyrosine kinase (Syk) [81]. Additionally, SCFAs prevented the development of intestinal inflammation via inhibiting dual-specificity phosphatase 6 (DUSP6) through the up-regulation of microRNA-145 (miR-145) by decreasing the CCAAT enhancer-binding protein beta (CEBPB) expression, and they also improved the disease activity index of LPS-treated mice [82].

In short, SCFAs, especially butyrate, generally showed protective effects on IBDs. The mechanisms of action involved enhancing intestinal barrier function, regulating immune system, and reducing inflammation.

### 2.8. Effects on Constipation

Constipation is a multifactorial intestinal disorder with high incidence and difficult treatment, leading to a serious decline in quality of life [83]. Studies have identified SCFAs as important regulatory factors in constipation. For example, an epidemiological study found that acetate, propionate, and butyrate were negatively correlated with the severity of constipation [84]. Another study with analysis of human feces found that the concentrations of SCFAs, particularly butyrate, in the feces of constipated patients, were lower than those of healthy people [85]. Furthermore, studies showed that acetic acid and butyric acid, but not propionic acid, could relieve constipation [86,87]. A study indicated that acetic acid increased water content of feces and small intestinal transit rate, and butyric acid decreased the transit time through the gut [86]. Besides, butyrate attenuated constipation symptoms by promoting the secretion of colonic hormones and maintaining intestinal barrier integrity [85]. Moreover, butyrate promoted defecation, improved intestinal mobility, and promoted Cajal cells proliferation via activating the AKT-NF-κB signaling pathway [88].

In brief, SCFAs, especially butyrate, play a key role in regulating colonic motility and might be a novel therapy of constipation.

### 2.9. Neuroprotective Activity

The gut microbiota and brain communicate with each other in a variety of ways and involve microbial metabolites, such as SCFAs [89]. Recent studies have shown that SCFAs are important mediators of the microbiota–gut–brain axis and play a crucial role in regulating the physiology and behavior of the central nervous system [90,91]. A study based on 116 Polish women suggested that depressive women had significantly lower levels of acetate and propionate compared with healthy individuals [92]. A study showed that acetate improved cognitive impairment, decreased the cluster of differentiation 11b (CD11b, a tight junction protein) level, and suppressed neuroinflammation in the Alzheimer’s disease model mice. For BV2 cells, acetate exerted anti-neuroinflammatory effects via inhibiting the phosphorylation of NF-κB, p65, ERK, and JNK; decreasing cyclooxygenase-2 (COX-2) and IL-1β levels; and increasing the GPR41 level [93].

In summary, some SCFAs, such as acetate, may help to prevent and manage neurodegenerative and neuropsychiatric diseases.

### 2.10. Anticancer Activity

Cancer is a serious public health problem worldwide, and cancer cells exhibit specific characteristics compared with normal cells, such as infinite proliferation, weak adhesion, and strong agglutination [94,95]. SCFAs could exert anticancer activities through different pathways. For example, SCFAs inhibited proliferation of human cervical cancer HeLa cells via down-regulating free fatty acid receptor 2 (FFAR2) expression [96]. Another study showed that acetate and propionate inhibited the carcinogenesis process of colorectal cancer via inhibiting cancer cell proliferation and inducing cell cycle arrest [97]. The SCFAs decreased the incidence and size of colitis-associated colorectal tumor in azoxymethane/dextran sodium sulfate-treated mice by improving colon inflammation and decreasing cell proliferation [98]. Besides, propionate and butyrate reduced the lung metastasis of melanoma cells by increasing the expression of chemokine (C-C motif) ligand 20 (CCL20) in lung endothelial cells and reducing the recruitment of Th17 cells [99]. It was reported that valeric acid suppressed colony formation, migration, and invasion of liver cancer cells in vitro, and suppressed hepatocellular carcinoma development in vivo, as well as improving the survival rate of mice with liver cancer [100]. Additionally, pentanoate and butyrate enhanced the effect of cancer immunotherapy by enhancing cytotoxic T lymphocytes and chimeric antigen receptor T cells via epigenetic and metabolic reprogramming [101]. Furthermore, the anticancer mechanisms of SCFAs also involved inducing apoptosis, activating autophagy, and modulating hematopoiesis [18,102,103].

In short, SCFAs could be a potential agent against several cancers, such as cervical, colorectal, melanoma, and liver cancers. The mechanisms of action mainly included inhibiting cancer cell proliferation, arresting cell cycle, decreasing inflammation, reducing metastasis, and enhancing effect of immunotherapy.

### 2.11. Anti-Bacterial Activity

SCFAs have been shown to inhibit bacterial growth and viability. A study showed that butyrate could enhance the antimicrobial activity of macrophages without tissue damaging inflammation [104]. Moreover, bacterial infection is one of the main causes of diarrhea. Enterotoxin-producing *Bacillus cereus*, *Clostridium difficile*, *Clostridium perfringens*, *Escherichia coli*, *Staphylococcus aureus,* and *Vibrio cholerae* are the major pathogenic bacteria that secrete highly toxic proteins, which could induce diarrhea [105]. The SCFAs could inhibit these bacteria, decrease enterotoxin cytotoxicity via mimicking the structure of toxin receptors and inhibiting toxin adherence to host cells, and promote the production of beneficial microbiome [105]. In addition, SCFAs could inhibit other foodborne pathogenic bacteria, such as *Campylobacter*, *Salmonella,* and *Shigella* in a pH-, dose-, and complexity-dependent manner [106]. Furthermore, SCFAs could be used to prevent food spoilage due to its antimicrobial activity. The evidence showed that repeated treatments with acetic acid vapors could preserve table grapes’ fruit quality, which was safer compared with SO_2_ [107].

Overall, SCFAs could prevent and treat bacterial diarrhea via inhibiting the pathogenicity of enteropathogens and decreasing enterotoxin toxicity. Moreover, SCFAs could also play a vital role in prevention of food spoilage.

### 2.12. Effects of SCFAs on Other Diseases

SCFAs also have other health benefits besides the bioactivities mentioned above. For example, a study found that propionate could alleviate mitochondrial dysfunction, oxidative stress, and apoptosis induced by free fatty acids via up-regulating peroxisome proliferator-activated receptor-gamma coactivator-1 alpha (PGC-1α) [108]. Besides, SCFAs could contribute to infant sleep. A study indicated that the higher the proportion of propionate in total fecal SCFAs, the longer the infant at 7 and 8 months of age slept uninterrupted [109]. Moreover, SCFAs could mediate the effects of intestinal microbiota on the metabolism and function of skeletal muscle. It was reported that SCFAs could regulate lipid, carbohydrate, and protein metabolism in skeletal muscle tissues, and they could also increase skeletal muscle mass retention. The mechanisms of action might be related to the activation of AMPK, PPAR-δ, and PGC-1α, as well as the inhibition of HDACs [110]. Moreover, acetate and propionate might contribute to maintaining a positive balance of bone turnover by up-regulating differentiation in primary osteoblasts [111]. Another study suggested that propionate and butyrate increased systemic bone mass under steady-state conditions via inducing the reprogramming of osteoclasts metabolism, enhancing glycolysis, expensing oxidative phosphorylation, and down-regulating several osteoclast genes, such as *TRAF6* and *NFATc1* [112]. Besides, SCFAs could also prevent bone loss after menopause [112]. Additionally, acetate, propionate, and butyrate synergistically alleviated rheumatoid arthritis by regulating B cells differentiation via FFAR2 receptors [113]. Furthermore, high concentrations of acetate and butyrate could suppress periodontal disease by decreasing the accumulation of neutrophil granulocytes in the gingival pockets via binding FFAR2 and triggering neutrophil Ca^2+^ signaling and chemotaxis [114].

In brief, SCFAs also exerted beneficial effects on sleep, skeletal muscle, bone loss, arthritis, and periodontal disease.

**Table 1:** Health benefits of SCFAs from epidemiological studies.

Study Type	Individuals	Outcomes	Ref.
***Anti-obesity***			
Prospective study	1585 singleton late preterm or full-term born	Human milk SCFAs could prevent excess weight gain in infants	[31]
***Hepatoprotective activity***			
Prospective study	49 patients with cirrhosis	SCFAs were negatively correlated with cirrhosis disease severity	[54]
***Effects on constipation***			
Cohort study	30 patients with ascending colon cancer and 90 patients with mixed refractory constipation	SCFAs were negatively correlated with the severity of constipation	[84]
***Neuroprotective activity***			
Cross-sectional study	116 women	Depressive women had lower levels of acetate and propionate	[92]

Abbreviation: SCFAs, short-chain fatty acids.

**Table 2:** Health benefits and molecular mechanisms of SCFAs from preclinical studies.

SCFAs Species	Study Type	Subjects	Doses	Effects and Mechanisms	Ref.
***Anti-inflammation***					
Acetate	In vivo	C57BL/6 mice and C57BL6 GFP Het	150 mM in drinking water	Induce caspase-dependent apoptosis of neutrophils; Decrease the activity of NF-κB; Enhance production of IL-10, TGF-β, and annexin A1.	[22]
Propionate	In vitro	HPMECs	0.6 mM	Accelerate Nrf2 nuclear translocation; Protect cells and promote angiogenesis; Reduce inflammatory response via NF-κB pathway.	[23]
Propionate	In vivo	C57BL/6J and Nrf2^−/−^ mice	1.2 mg/g i.p.	Reduce pulmonary inflammation and oxidative stress.	[23]
Propionate	In vivo	BALB/c and C57BL/6 mice	150 mM in drinking water	Interfere with the production and migration of inflammatory mediators.	[24]
Butyrate	In vivo	ICR mice	200 mg/kg i.p.	Up-regulate the IL-10 in septic shock.	[21]
Butyrate	In vitro	RAW 264.7 cells	100 μM	Down-regulate the IL-6 and IL-1β; Increase the IL-10.	[21]
Propionate; Butyrate	In vitro	THP-1 cells	10 µM	Inhibit the expressions of IL-4, IL-6, and ROS; Enhance the expressions of IL-10 and IFN-γ.	[20]
***Immunoregulation***					
Butyrate	In vivo	C57BL/6J mice	200 mM in drinking water	Promote IL-22 production by CD4^+^ T cells and ILCs.	[28]
Acetate; Propionate; Butyrate	In vitro	CD4^+^ T cells	10 mM acetate; 0.5 mM propionate; 0.5 mM butyrate	Promote CD4^+^ T cell and ILC production of IL-22 through GPR41 and HDAC inhibition.	[28]
Acetate; Propionate; Butyrate	In vitro	Synovial fibroblasts	250 µM propionate or the mixture (300 µM acetate, 100 µM propionate, 100 µM butyrate)	Interfere with arthritogenic properties of synovial fibroblasts; Induce cellular senescence.	[24]
Acetate; Propionate; Butyrate	In vivo	C57BL/6J and C.129-IL4tm1Lky/J (4get) mice	40 mM butyrate, 67.5 mM acetate and 25.9 mM propionate in drinking water	Regulate T cells and DC activities; Reduce the production of IL4-producing CD4^+^ T cells; Decrease circulating IgE level.	[27]
Acetate; Propionate; Butyrate; Pentanoate	In vitro	Splenic B cells isolated from C57BL/6J mice	0.5 mM of NaAc, NaPr, NaBu or NaPe	Promote B10 cell generation; Enhance B10 cell function.	[29]
Acetate; Propionate; Butyrate; Pentanoate	In vivo	C57BL/6J mice	150 mM acetate, propionate, butyrate or pentanoate in drinking water	Promote B10 cell generation via activation of GPCR.	[29]
***Anti-obesity***					
Propionate	In vitro	YAMC cells	5 mM	Repress the TG accumulation via modulating the expression levels of PPARα-responsive genes.	[36]
Acetate; Propionate; Butyrate	In vitro	3T3-L1 cells	6.4 mM acetic acid; 3.2 mM propionic acid or 0.4 mM butyric acid	Accelerate the 3T3-L1 adipocyte differentiation; Promote lipid accumulation via modulation of the expression of LPL, adipocyte FABP4, FATP4, and FAS.	[115]
Acetate; Propionate; Butyrate	In vivo	C57BL/6J mice	5% acetate, propionate, or butyrate in the diet	Protect against high-fat diet-induced obesity; Suppress hepatic weight and lipid synthesis.	[37]
Acetate; Propionate; Butyrate	In vivo	C57BL/6 mice	6 mmol/kg acetate; 6 mmol/kg propionate; 1–6 mmol/kg butyrate, 10 mL/kg i.p.	Activate vagal afferent neurons.	[32]
Acetate; Propionate; Butyrate	In vivo	C57BL/6J mice	5% sodium acetate; 5% sodium propionate or 5% sodium butyrate in a high-fat diet	Reduce appetite and fat accumulation via modulating relevant genes and hormones; Regulate the expressions of several mRNA.	[34]
Acetate; Propionate; Butyrate	In vivo	Barrows (Duroc × Landrace × Yorkshire)	0.1% acetate; 0.1% propionate; 0.1% butyrate; 0.1% mixed SCFAs (3:1:1) in diet supplement	Increase the concentrations of the serum GLP-1, PYY and leptin to regulate the appetite; Down-regulate of the mRNA expressions of FAS, ACC, and SREBP-1C to participate the de novo synthesis of fatty acids; Enhance the mRNA expressions of LIPE and CPT-1α to participate in fatty acids oxidation.	[33]
Acetate; Propionate; Butyrate	In vivo	Long–Evans rats	M_NaAc_:M_NaPr_:M_NaBu_ = 60:20:20, dissolve in 0.1 M PBS, 500 mg/kg i.p.	Suppress the de novo lipogenesis by reducing mRNA expression of hepatic Acc1.	[35]
***Cardiovascular protection***					
Acetate	In vivo	SD rats	200 mmol/L magnesium acetate in drinking water	Up-regulate SCFAs receptors Olfr78, GPR41, and GPR43 to keep the balance of vasoconstriction and vasodilation shifts.	[47]
Propionate	In vivo	NMRI and ApoE^−/−^ mice	200 mmol/L in drinking water	Reduce blood pressure; Attenuate cardiac hypertrophy, fibrosis, and vascular dysfunction.	[48]
Propionate	In vivo	ApoE^−/−^ mice	200 mg/kg i.g.	Reduce intestinal cholesterol absorption and aortic atherosclerotic lesion area; Increase levels of regulatory T-cell numbers and IL-10; Reduce the levels of NPC1l1.	[44]
Butyrate	In vitro	Caco-2 cells	0, 0.1, 1 and 10 mmol/L	Inhibit cholesterol absorption; Reduce the levels of NPC1l1; Increase the levels of ABCG5 and ABCG8.	[43]
Butyrate	In vivo	SD rats	200 mmol/L in drinking water	Improves myocardial I/R injury via gut-brain neural circuit.	[49]
Acetate; Propionate; Butyrate	In vivo	C57BL/6J mice	100 mmol/L in drinking water	Reduce blood pressure.	[46]
Acetate; Propionate; Butyrate; valerate	In vivo	Golden Syrian hamsters	0.5 mol/kg sodium acetate, sodium propionate, sodium butyrate, and valeric acid in high-cholesterol diet	Enhance fecal excretion of bile acids; Up-regulate the gene expressions of SREBP2, LDLR, and CYP7A1 in the liver.	[45]
***Hepatoprotection***					
Acetate	In vivo	C57BL/6 mice	200µL LITA-Rhd i.p.	Decrease lipid accumulation; Improve hepatic function; Increase mitochondrial efficiency.	[39]
Propionate	In vitro	HepG2 cells	0.2, 0.4, 0.8 mM	Enhance hepatic function; Alleviate ethanol-induced hepatic steatosis.	[56]
Propionate	In vivo	C57BL/6J mice	100 or 200 mM in the diet	Prevent ethanol-induced loss of hepatic function; Alleviate ethanol-induced hepatic steatosis.	[56]
Acetate; Propionate; Butyrate	In vitro	BRL 3A cell	10 mM NaAc, 5 mM NaPr, 2.5 Mm NaBu or 10 mM mixture (M_NaAc_:M_NaPr_:M_NaBu_ = 3:1:1)	Reduce the production of ROS and MDA; Activate AMPK and PPAR signaling pathways; Down-regulate the expression of genes related to lipid synthesis.	[59]
Acetate; Propionate; Butyrate	In vitro	human-iPSC-derived liver organoids	1 μM acetate; 1 μM propionate; 1 μM butyrate	Increase the expression of CYP3A4 and ALB.	[63]
***Anti-diabetes***					
Propionate	In vitro	HepG2 cells	0, 0.25, 0.5 mM	Suppress gluconeogenesis by down-regulation of gluconeogenic enzymes; Suppress hepatic gluconeogenesis by activating AMPK; Activate AMPK via Ca^2+^/CaMKKβ pathway.	[70]
Acetate; Propionate	In vivo	C3H/HeOuJ mice	5% SCFA (Ac:Pr, 2.5:1 or Ac:Pr, 1:2.5) in the diet	Attenuate high-fat diet-induced insulin resistance.	[38]
Acetate; Propionate; Butyrate	In vivo	C57BL/6 WT and IL22 KO mice	67.5 mM acetate, 40 mM butyrate and 25.9 mM propionate in drinking water	Prevent type 1 diabetes; Promote development of regulatory T cells.	[116]
Acetate; Propionate; Butyrate	In vivo	C57BL/6 mice	Acetate (5% w/w of diet), propionate (10% w/w of diet), butyrate (10% w/w of diet), acetate + propionate (5% + 10% w/w of diet)	Improve insulin sensitivity.	[67]
***Prevention and management of inflammatory bowel disease***					
Propionate	In vivo	WT C57BL / 6 mice	200 mM in drinking water	Promote intestinal epithelial cell migration; Increase cell speed and persistence.	[74]
Butyrate	In vivo	WT C57BL/6J mice	200 mM in drinking water	promote IL-22; Protect the intestines from *Citrobacter rodentium* infection.	[28]
Butyrate	In vitro	ICR mice crypt	100μM	Elicit α-defensin secretion by Paneth cells; Improve enteric innate immunity through potent microbicidal activities.	[79]
Acetate; Propionate; Butyrate	In vitro	Sheep ruminal tissue	60 mM NaAc, 30 mM NaPr and 10 mM NaBu	Release protons; Induce subacute ruminal acidosis.	[117]
Acetate; Propionate; Butyrate	In vitro	Caco-2 cells	acetate (12.5, 25, and 50 mM), butyrate (5, 10, and 20 mM) and propionate (5, 10, and 20 mM)	Increase the level of Hspa1a expression. Up-regulate HSP70; phosphorylate HSP1.	[76]
Acetate; Propionate; Butyrate	In vitro	Caco-2 and T84 cells	Acetate: 0–20 mM, propionate: 0–10 mM, butyrate: 0–2.5 mM	Reduce IL-8 and IL-6 expression levels; Reduce the activation of NF-κB, ERK, p38 MAPK, JNK, and Syk.	[81]
Acetate; Propionate; Butyrate	In vitro	MODE-K and MC38 cell lines	A mixture of 0.5 mM acetate, 0.01 mM propionate, and 0.01 mM butyrate	Inhibit DUSP6 by up-regulating miR-145 through decreasing the CEBPB expression.	[82]
Acetate; Propionate; Butyrate	In vitro	Caco-2 cells	0.5 mM acetate, 0.01 mM butyrate, 0.01 mM propionate	Increase TER; Improve the formation of tight junction; Inhibit the activation of NLRP3 inflammasome and autophagy induced by LPS.	[78]
Acetate; Propionate; Butyrate	In vivo	C57BL/6J mice	200 mM propionate, 200 mM acetate or 100 mM butyrate in the drinking water	Repress IL-17- and IL-22-producing γδ T cells; Reduce IL-17 production by γδ T cells by inhibiting HDAC.	[80]
Acetate; Propionate; Butyrate	In vivo	C57BL/6J mice	25 mM propionate, 40 mM butyrate and 67.5 mM acetate in drinking water	Inhibit DUSP6 via up-regulating miR-145 by suppressing CEBPB; Improve DAI.	[82]
***Prevention and management of constipation***					
Butyrate	In vitro	ICCs	0, 0.00005, 0.0005, 0.005, 0.05 and 0.5 mmol/L	Promote mouse ICC proliferation by activating AKT/NF-κB signaling.	[88]
Butyrate	In vivo	Kunming mice	1.1% in the drinking water	Promote defecation; Improve intestinal mobility; Activate the AKT-NF-κB signaling pathway.	[88]
Acetate; Propionate; Butyrate	In vivo	BALB/c mice	Diets supplemented with 150 g/kg of either acetylated starch, propylated starch, butylated starch	Acetylated starch and butylated starch relieve constipation; Acetic acid increases WCF and SITR; Butyric acid decreases the transit time through the gut.	[86]
***Neuroprotection***					
Acetate	In vitro	BV2 cells	1200 μM	Improve cognitive impairment; Decrease the CD11b level; Suppress neuroinflammation.	[93]
Acetate	In vivo	APP/PS1 transgenic and matched WT mice	1.5 g/kg i.g.	Inhibit the phosphorylation of NF-κB p65, ERK, and JNK; Decrease COX-2 and IL-1β levels; Increase GPR41 level.	[93]
Propionate	In vivo	Western albino rats	75 mg/kg or 250 mg/kg i.g.	Increase the levels of IFN-γ and caspase-3; Decrease levels of nor-adrenaline, dopamine, and 5-HT.	[118]
Propionate	In vivo	Western albino rats	75 mg/kg or 250 mg/kg i.g.	Increase the levels of glutamate and the glutamate/glutamine ratio; Decrease GABA, glutamine, and the GABA/glutamate ratio.	[119]
***Anticancer***					
Valerate	In vitro	Hep3B, SNU-449, HepG2, THLE-3, MCF-7, MDA-MB-231, MCF-10A, A549, U-87 and A172, HeLa, DU145, and HL-60 cells	0.5, 1, 2, 4, 8 mM	Suppress colony formation, migration, and invasion of liver cancer cells; Suppresses 3D spheroid formation of liver cancer cells.	[100]
Valerate	In vivo	Athymic nude mice	100 mg/kg tail injection	Suppress HCC development; Improve the survival rate.	[100]
Butyrate; Propionate	In vivo	C57BL/6 mice	300 mg/kg acetate, 150 mg/kg propionate or 88 mg/kg butyrate i.p.	Increase the expression of CCL20; Reduce the recruitment of Th17 cells; Inhibit the lung metastasis of melanoma cells.	[99]
Acetate; Butyrate; Propionate	In vivo	BALB/c mice	67.5 mM acetate, 40 mM butyrate and 25.9 mM propionate in drinking water	Decrease cell proliferation.	[98]
Acetate; Propionate; Butyrate Pentanoate	In vivo	CD45.1 WT; CD45.2 WT; CD45.1 OT-I; CD45.2 Ffar2^−/−^ Ffar3^−/−^ mice; CD45.2 FIR × tiger; Rag1^−/−^ mice	0.5, 1.0, 2.5 mM	Increase the anticancer activity of cytotoxic T lymphocytes and chimeric antigen receptor T cells via metabolic and epigenetic reprogramming.	[101]
***Prevention and management of arthritis***					
Acetate; Propionate; Butyrate	In vivo	WT C57BL/6J mice and DBA/1J mice	150 mM acetate, propionate or butyrate in drinking water	Increase systemic bone mass via inducing the reprogramming of osteoclasts metabolism, enhancing glycolysis, and down-regulating *TRAF6* and *NFATc1*; Prevent bone loss after menopause; Alleviate arthritis.	[112]
Acetate; Propionate; Butyrate	In vivo	DBA/1JGpt, *Ffar2*^fl/fl^ and *Ffar2*^fl/fl^/CD19-Cre mice	150 mM acetate, propionate or butyrate in drinking water	Synergistic treatment of CIA; Regulate B cell differentiation via FFA2 receptors; Suppress the inflammatory response.	[113]

Abbreviation: ABCG5, ATP-binding cassette transporters G5; ABCG8, ATP-binding cassette transporters G8; Ac, Acetate; Acc1, acetyl-CoA carboxylase-1; CaMKKβ, Ca^2+^-calmodulin-dependent protein kinase β; CCL20, chemokine (C-C motif) ligand 20; CEBPB, CCAAT enhancer-binding protein beta; CIA, collagen-induced arthritis; COX-2, cyclooxygenase-2; CPT-1α, carnitine palmitoyltransferase-1α; CYP7A1, cholesterol 7 alpha-hydroxylase; DAI, disease activity index; DC, dendritic cells; DUSP6, dual-specificity phosphatase 6; ERK, extracellular signal-regulated kinase; FABP4, fatty acid binding protein 4; FATP4, fatty acid transporter protein 4; FAS, fatty acid synthase; GABA, gamma amino butyric acid; GPR41, G-protein-coupled receptor 41; HAMS, High-amylose maize starch; HCC, hepatocellular carcinoma; HPMECs, human primary pulmonary microvascular endothelial cells; HSP, heat shock protein; ICCs, interstitial cells of Cajal; IFN-γ, Interferon-γ; Ig, immunoglobulin; i.g., intragastric administration; IL, interleukin; i.p., intraperitoneal injection; iPSC, induced pluripotent stem cell; I/R, ischemia/reperfusion; JNK, c-Jun N-terminal kinase; LDLR, low-density lipoprotein receptor; LIPE, lipase hormone sensitive; LPL, lipoprotein lipase; LPS, lipopolysaccharide; LITA, liposome-encapsulated acetate; MAPK, mitogen-activated protein kinase; miR, microRNA-145; NF-κB, nuclear factor-kappa B; NGNs, nodose ganglion neurons; NPC1l1, Niemann-Pick C1-like 1; NTS, nucleus tractus solitaries; OCFA, odd-chain fatty acids; Pr, Propionate; ROS, reactive oxygen species; SD, Sprague–Dawley; SITR, small intestinal transit rate; SREBP2, sterol-regulatory-element-binding protein 2; Syk, spleen tyrosine kinase; TER, transepithelial electrical resistance; TG, triglyceride; TGF-β, transforming growth factor-β; Th17, T helper 17; TNF, tumor necrosis factor; WCF, water content of feces; WT, wide type; YAMC, young adult mouse colon.

**Table 3:** Health benefits of SCFAs from clinical studies.

SCFAs Species	STUDY TYPE	Individuals	Administration Methods	Outcomes	Ref.
***Anti-obesity***					
Propionate	Single-blind crossover RCT	20 healthy men	Inulin-propionate ester (10 g/day) for 24 weeks	Reduce anticipatory reward responses in the human striatum to high-energy foods	[40]
***Cardio-protective activity***					
Propionate	Double-blind RCT	62 participants	Calcium-Propionate (500 mg, twice daily) for 8 weeks	Reduce levels of LDL and non-high-density lipoprotein cholesterol	[44]
***Anti-diabetic activity***					
SCFAs	RCT	29 overweight/obese individuals	Med-D intervention for 8 weeks	Increase plasma butyric acid Improve postprandial glucose metabolism and insulin sensitivity	[72]

Abbreviations: LDL, low-density lipoprotein; Med-D, Mediterranean diet; RCT, Randomized controlled trial; SCFAs, short-chain fatty acids.

## 3. The Side Effects of SCFAs

Although most studies showed that SCFAs exerted health benefits, several studies found that some SCFAs could be useless or exhibit side effects in some conditions. Although SCFAs from inulin fermentation had potential to prevent or treat type 1 diabetes, direct SCFAs oral administration alone did not provide a significant impact on the diabetes, which might be attributed to oral administration did not allow for enough SCFAs to reach the site of action (cecum and colon) [116]. Moreover, a meta-analysis including 44 randomized controlled trial studies found that acetate, propionate, butyrate, and the mixed SCFAs had no effect on insulin and blood glucose in humans [120]. Another study showed that SCFAs accelerated the 3T3-L1 adipocyte differentiation and promoted lipid accumulation via modulating the expression of fatty acid metabolism-related enzymes in vitro, such as lipoprotein lipase, adipocyte fatty acid-binding protein 4, fatty acid transporter protein 4, and fatty acid synthase [115]. Additionally, a study suggested that, for those who had NAFLD and poor metabolic health, the gut-derived acetate could provide an extra precursor for de novo lipogenesis in the liver, which could increase hepatic lipid accumulation [15]. In a multi-organ model of ulcerative colitis (UC) ex vivo, SCFAs improved UC severity via increasing the production of ketone bodies, glycolysis, and lipogenesis, and significantly decreased innate immune activation of UC gut, but SCFAs also induced gut barrier disruption through metabolic reprograming during acute T cell-mediated inflammation [121]. Furthermore, some SCFAs could induce brain neurochemistry impairment. For example, an epidemiological study found that depressive women had higher concentrations of isocaproic acid than healthy subjects [92]. Another study showed that propionate caused brain neurochemistry impairment by increasing the levels of IFN-γ and caspase-3, as well as decreasing nor-adrenaline, dopamine, and 5-HT [118]. Moreover, propionate increased the level of glutamate and the glutamate/glutamine ratio, and decreased gamma amino butyric acid (GABA), glutamine and the GABA/glutamate ratio, which showed characteristics of autism [119].

In short, different SCFA types might exert different effects, and the bioactivities of SCFAs might depend on the health condition of hosts. When this section was compared with previous sections, the results from epidemiological studies could be inconsistent. In the future, more high-quality, large sample epidemiological studies are needed to verify the effects of SCFAs on humans.

## 4. Conclusions

SCFAs show various bioactivities, such as anti-inflammatory and immunoregulatory effects, as well as preventive and therapeutic effects on several diseases, including obesity, cardiovascular disease, liver disease, diabetes mellitus, IBDs, diarrhea, constipation, neurodegenerative diseases, neuropsychiatric diseases, cancers, arthritis and periodontal disease, and so on. Moreover, the underlying mechanisms are diverse, among which NF-κB, Nrf2, GPR41, and HDAC signaling pathways are highlighted. Furthermore, it is worth noting that, due to differences in SCFAs types and concentrations as well as health condition of hosts, some SCFAs could exhibit a double-sided effect. Therefore, in the application of SCFAs, attention should be paid to the selection of SCFA type, as well as the influence of their concentration and body condition. In the future, more bioactivities of SCFAs should be evaluated, and their mechanisms of action should be further studied. Furthermore, because most of the results comes from preclinical models, more clinical trials should be carried out to verify these potential effects of SCFAs on human beings. This paper is helpful for supporting people eating more dietary fiber, and for some SCFAs and dietary fiber to be developed into functional food to prevent and manage several diseases.

## Data Availability

The data presented in this study are available on request from the corresponding author.
